# Motivations of nursing students regarding their educational preparation for mental health nursing in Australia and the United Kingdom: a survey evaluation

**DOI:** 10.1186/s12912-015-0084-8

**Published:** 2015-05-10

**Authors:** Karen-leigh Edward, Philip Warelow, Stephen Hemingway, Gylo Hercelinskyj, Anthony Welch, Sue McAndrew, John Stephenson

**Affiliations:** Nursing Research, Faculty of Health Sciences, School of Nursing, Midwifery and Paramedicine, Australian Catholic University, St Patrick’s Campus, Melbourne, Locked Bag 4115, FITZROY, MDC 3065 Australia; Nursing Research Unit, St Vincent’s Private Hospital Melbourne, 59-61 Victoria Parade, Fitzroy, VIC 3065 Australia; School of Human and Health Sciences, University of Huddersfield, Queensgate, Huddersfield HD1 3DH UK; Faculty of Health, Federation University, Mt. Helen Campus, PO Box 663, Ballarat, VIC 3353 Australia; Mental Health, School of Human and Health Sciences, University of Huddersfield, Queensgate, Huddersfield HD1 3DH UK; Faculty of Health Sciences, School of Nursing, Midwifery and Paramedicine, Australian Catholic University, St Patrick’s Campus, Melbourne, Locked Bag 4115 FITZROY, MDC 3065 Australia; Mental Health Nursing, Assistant Dean Community Engagement, School of Nursing and Midwifery, Faculty of Sciences, Engineering and Health, CQUniversity Australia, 90 Goodchap Street, (PO Box 1128), North Rockhampton, Noosaville BC QLD 4566 Australia; School of Nursing and Midwifery, Mary Seacole Building, Greater Manchester, Salford M6 6PU, UK; Biomedical Statistics, School of Human and Health Sciences, University of Huddersfield, Queensgate, Huddersfield HD1 3DH UK

**Keywords:** Curriculum, Mental health nursing, Motivation, Nursing education, Psychiatric nursing

## Abstract

**Background:**

There has been much debate by both academics and clinical agencies about the motivations and abilities of nurse graduates to work in mental health nursing. The aim of this study was to recruit student nurses from a dedicated mental health nursing program in the United Kingdom (UK) and a comprehensive nursing program in Australia and illuminate their motivations towards considering mental health nursing as a career choice.

**Methods:**

This study comprised of two UK and four Australian Schools of Nursing within Universities. A 12 item survey was developed for the purpose of this study and was checked for face validity by experienced mental health nurses. Convenience sampling was used and 395 responses were received.

**Results:**

The comprehensive program represented by the Australian sample, revealed a third of respondents indicated that mental health nursing was definitely not a career option, while only 8 % of the UK specialised program reported mental health nursing was not seven for them. In both groups a higher level of motivation to work in mental health emanated from personal experience and/or work experience/exposure to mental health care.

**Conclusions:**

A greater focus on clinical exposure in comprehensive programs could enhance professional experience needed to increase student motivations for mental health nursing.

## Background

Nursing education programs in the United Kingdom (UK) and Australia are distinctly different in the way they prepare students for graduate clinical practice. While the UK maintains a discrete field for mental health nursing [1] Australia has adopted a comprehensive approach to nurse education [2]. Comprehensive programs were designed to provide students with a sound knowledge and skill base for beginning clinical practice in a range of different nursing eventualities [3]. There has been considerable debate by both academics and clinical agencies about the motivations and abilities of nurse graduates from comprehensive nurse programs to work in mental health resulting in calls by some mental health nursing professionals for the reintroduction of a discrete undergraduate mental health nursing (MHN) program [4]. However, small but encouraging outcomes related to recruitment to mental health nursing have been seen in students who had enrolled in a mental health major undergraduate degree [5]. It is widely accepted that nursing as a career is viewed favourably by society in that it is perceived as offering job security, mobility and career variety. While the same cannot be said for particular speciality streams of nursing, such as mental health nursing, in the UK, recruitment into mental health nursing degree programs is stable, with all universities filling the available places [1, 6]. Recent evidence demonstrates that MHN as a career is generally not a popular choice for undergraduate nurses enrolled in a comprehensive degree program [2]. Therefore the decision to survey students from each program type was made to gain insights into students’ motivations toward MHN as a career choice. While this project cannot compare the two programs due to the different curriculum designs, the information can offer insights into the motivations of students with regards to choosing MHN as a career direction. This is an important point of discovery since making good career choices for individuals includes such information as clarifying underlying values and interests, managing uncertainty and using experiences and/or knowledge to develop career plans with possible alternatives if career pathways should not go according to plan.

## Methods

### Participating sites

The study was comprised of two UK and four Australian Schools of Nursing within Universities. The university course structure in the UK offered discipline specific (field) entry level mental health nursing programs, while in Australia the education for nurses was offered in more broad based comprehensive programs. This difference in nursing preparation is central to the choice of these countries; the UK hypothesising that specialisation preparation would yield a student who was highly motivated to enter MHN. It is not known however what motivates student nurses towards mental health in comprehensive nursing programs and whether similarities may exist between the two approaches to nurse education.

### Study design and ethical considerations

Employing convenience sampling method, this study used a survey to collect data. This design was used for a number of reasons including affordability (surveys are inexpensive and can be administered online increasing accessibility), flexibility (questions were tailored to address the specific hypothesis) and dependability (surveys allows participants to remain anonymous increasing the chances of candid and valid responses). The survey comprised nine closed and three open ended/extended response questions that comprised of yes/no type questions, and multiple choices type questions. Frequencies were used to report the survey result information. There were questions related to demographic information, previous personal or work experience prior to commencing undergraduate studies, and intended career pathway on completion of the course. The survey was developed for the purpose of this study by six experienced mental health nurses involved in the study and located in both countries. Questions were developed in consideration of mental health nursing as a career choice for students. The survey was examined for face and content validity by experienced mental health nurses. Participating students received the survey as either a hard copy or via an on-line link. Ethics approval was obtained from six individual University Human Research and Ethics Committees (HREC). These were - Australian Catholic University HREC, University of Canberra HREC, University of Ballarat HREC, Queensland University of Technology HREC, University of Huddersfield HREC and Salford University HREC. No identifying material has been used in the results of this study and only aggregated data are presented.

### Participants

#### Inclusion criteria

The inclusion criteria for participation in this study were:

*In Australia*, students were required to be currently enrolled in the Bachelor of Nursing (BN) Degree or a double degree (such as a Bachelor of Nursing and Bachelor of Midwifery combined degree) with a BN. All Australian participants were enrolled in the second or third year of the program, and had undertaken the mental health aspect of the course.

*In the UK*, students were enrolled in diploma or degree programs leading to registration as a Mental Health Nurse (MHN). Participants were sought from the first year cohort as the common foundation is where the participants undertook the generic course content before entering the specific mental health part of the programme, thus allowing for a ‘best fit’ comparison to be made.

### Recruitment and data collection

As each university involved in the study had different academic and/or clinical placement schedules, the mode of inviting students to participate was tailored to meet the schedules of each university. Participants were informed of the study and invited to participate in one of three ways: (1) during their mental health class by one of the researchers, (2) by the student university email address or (3) by placing an invitation flyer on relevant electronic announcement systems for each university. The participant information sheet, consent form and survey for the study were available to students in either hard copy or electronic format. Data was collected between September 2011 and February 2012.

### Data analysis

Frequencies were generated where questions of the survey were analysed using the Statistical Package for the Social Sciences (Version 20.0) software program [7].

## Results

### Descriptive summary

A total of 395 responses were received from both countries, comprising 249 (63.0 %) from Australia and 146 (37.0 %) from the UK. The Australian participants represented three states of Australia while respondents in the UK sample were located in the North and North-West of England. With a potential participant pool of approximately 1200 students (about 760 from Australia and about 440 from UK), this represents a recruitment rate of 33 %.

Participants were asked to provide demographic information relating to age, gender, ethnicity, locality (distance of home from place of study) and level of prior education. Some demographic differences between the Australian and UK samples were observed. The proportion of participants stating their ethnicity as white was higher amongst Australian participants than British participants who had a higher proportion of participants stating their ethnicity as African. Other ethnicities were approximately equally represented in both countries. The mean age of participants in both countries was similar (see Table 1).

**Table 1:** Demographic information of participants

Characteristic	Frequency (% valid responses)		
	All respondents (n = 395)	Australia (n = 249)	UK (n = 146)
**Gender**			
Male	60 (15.2 %)	22 (8.9 %)	38 (26.0 %)
Female	335 (84.8 %)	225 (91.1 %)	108 (74.0 %)
**Age range**			
15-19	26 (6.6 %)	20 (8.1 %)	6 (4.1 %)
20-24	172 (43.5 %)	116 (47.0 %)	54 (37.0 %)
25-29	63 (15.9 %)	35 (14.2 %)	28 (19.2 %)
30-34	36 (9.1 %)	22 (8.9 %)	14 (9.6 %)
35-39	30 (7.6 %)	16 (6.5 %)	14 (9.6 %)
40+	68 (17.2 %)	38 (15.4 %)	30 (20.5)
**Ethnicity**			
White	321 (81.3 %)	209 (84.6 %)	110 (75.3 %)
Asian	17 (4.3 %)	14 (5.7 %)	3 (2.1 %)
African	28 (7.1 %)	5 (2.0 %)	23 (15.8 %)
Chinese	9 (2.3 %)	8 (3.2 %)	1 (0.7 %)
Other	20 (5.1 %)	9 (3.6 %)	7 (4.8 %)
**Locality**			
Local	294 (74.6 %)	157 (63.8 %)	136 (93.2 %)
National	92 (23.4 %)	81 (32.9 %)	10 (6.8 %)
International	8 (2.0 %)	8 (3.3 %)	0 (0.0 %)

### United Kingdom results

Prior work experience in health and previous nursing/social experience of mental health was more prevalent amongst the UK cohort. All UK respondents were enrolled in a diploma or nursing degree with a specialty in Mental Health. Of the UK cohort (n = 146) 54 % indicted they had previous experience of mental health issues (either personal or professional) and nearly 57 % of respondents indicated MHN teaching was the only focus of the degree content. Within the group of UK participants the main motivational factor for choosing or not choosing MHN as a career was based upon personal experience (48 %) and clinical placement (42 %). A small number of the respondents (8 %) indicated they would not choose MHN as a career after the degree (for reasons unknown).

### Australian results

The majority of the 249 Australian respondents were enrolled in a comprehensive nursing degree (in which University mental health education had been only delivered as a stand-alone subject(s) within the whole degree), and a small number were also enrolled in a midwifery degree. Approximately 61 % of the cohort had never had previous experience of mental health issues either personally or professionally. MHN teaching in the degree program was indicated as being predominately a standalone subject (53.8 %) or integrated throughout the course in various ways. The main motivating factor for considering MHN as a career was indicated by 32 % responding to this question stating this would be an option. However, 24 % of respondents indicated personal experience would be the major motivational factor for choosing or not choosing MHN as a career. A third of respondents indicated they were unsure whether they would choose MHN as a career and a third indicated they would not choose MHN at all (33.8 %).

## Discussion

### Mental health nursing as a career option

The comprehensive program represented by the Australian sample revealed a third of respondents indicated MHN was definitely not a career option for them. This finding is similar to other studies in terms of the poor popularity of MHN as a career choice for nurse graduates of the comprehensive degree [2]. However, encouragingly a similar number of the Australian sample indicated they would either consider MHN as a choice of career or remained undecided. This is a more positive outcome than seen in other studies [2, 5]. When considering the Australian cohort who were enrolled in a comprehensive degree, the results of our study are similar to one undertaken with medical students [7]. In a study undertaken by Halder et al. [8] medical students were divided into distinct groups - those deciding on psychiatry before medical school, those deciding on psychiatry during medical school, and those interested in other fields. The study revealed there was little difference noted in the student ratings of lectures between those who chose psychiatry and those who did not. However, interestingly the study results suggest that enhancing the exposure/experience of mental health may improve recruitment to psychiatry. This finding is also similar for nursing students [5]. Mental health service delivery is often experienced as a different speciality to others in health due to issues such as stigma and uncertainty related to the professionals role [5, 9].

### Prior exposure to mental illness

In both the UK and Australian cohorts participating in this study personal and/or professional experience of mental health issues appeared to have had an impact on the decision making of students when considering their career options. This phenomenon has been observed in students in other studies who describe a desire to make a difference to *‘another’*. This altruistic desire appeared to emerge after exposure to the area of psychiatry when they are in their under graduate degree and can be expressed later as a career choice [9]. A desire to make a difference was also evident in other health disciplines such as allied health where altruistic motivations dominate career choice [10]. It is in this context that helping another may offer benefits for both the helper and the recipient [11, 12]. Immersion in mental health settings for students to develop or discover pro-social behaviours (behaviour intended to benefit another) may manifest into a possible desire to make a difference to people who live with mental illness.

### Career immersion and mentoring in mental health nursing

Enhancement of motivation for opting for MHN as a career option as revealed in the findings of this study suggests greater exposure or experience of mental illness through such activities as career immersion, mentorship, preceptorship or personal experience. Structured career immersion in mental health education, with an emphasis on the important role of mentors, may offer positive returns for nursing [13]. Career immersion has been used in some nursing settings to prepare students. For example a brief immersion practicum at a mental health facility where students were assigned to one specific setting for six consecutive weekdays (45 h), without the distraction of other classes, was undertaken due to declining clinical rotation availabilities. Students thought that their ability to form therapeutic relationships with consumers and integrate theory into clinical practice was enhanced through this immersion experience [14]. It has been suggested that such immersion may take various formats such as simulation experience as a person who is living with serious mental illness [15], simulated case scenarios using high fidelity simulations for mental health education [16] or 40 h per week for a full semester after completing theoretical content [17].

### Limitations

The limitations of the study relate to a purposive sample as compared to random selection. Potentially this could affect the generalisability of the findings if the sample was systematically different from the parent population; however, no evidence for selection bias or response bias was apparent. Another limitation was the use of a survey that was not tested for reliability; however it was determined to have face validity by expert MHN’s. In light of these limitations generalisations are prohibitive, nevertheless findings do highlight areas for further exploration, particularly ways to increase exposure of mental health education in comprehensive nursing curriculums.

## Conclusions

This study’s findings revealed that a personal experience of mental health (such as a relative with a mental illness or experience in working with people who experience mental illness) enhanced student motivation for opting for a career in mental health nursing in both the UK and the Australian programs. Career immersion, as undertaken in the specialty program, may offer a means whereby students enrolled in a comprehensive nursing degree have greater exposure to mental illness and thus an opportunity to develop or discover pro-social behaviours (such as a desire to make a difference to someone experiencing mental illness). Career immersions have been seen to work in both medical and nursing curriculums in recent times and may be developed in curriculums using a variety of techniques (such as simulation and block placements without the distraction of lectures). While it could be assumed that students enrolled in a specialised MHN program are potentially more motivated towards that specialisation than a comprehensively educated nurse the knowledge of student motivations in terms of making those career choices has remained unclear. This study’s findings suggest that these motivations are quite similar and in particular exposure to mental illness being an important influence for student nurses. Further examination of career immersion in mental health nursing and its impact on career choice however is warranted.

## Abbreviations

MHN: Mental Health Nursing
UK: United Kingdom
BN: Bachelor of Nursing
